# Blowflies are potential vector for avian influenza virus at enzootic area in Japan

**DOI:** 10.1038/s41598-024-61026-1

**Published:** 2024-05-04

**Authors:** Ryosuke Fujita, Takuji Tachi, Masato Hino, Kosuke Nagata, Masahiro Saiki, Mizue Inumaru, Yukiko Higa, Kentaro Itokawa, Nozomi Uemura, Ryo Matsumura, Izumi Kai, Kyoko Sawabe, Mutsuo Kobayashi, Haruhiko Isawa, Takahiro Kusakabe, Kazunori Matsuo, Shinji Kasai

**Affiliations:** 1 Laboratory of Sanitary Entomology, Faculty of Agriculture, Kyushu University, West-5 543, Motooka 744, Nishi-ku, Fukuoka, 819-0395 Japan; 2 Biosystematics Laboratory, Faculty of Social and Cultural Studies, Kyushu University, Fukuoka, Japan; 3 Hokkaido Research Organization, Sapporo, Japan; 4 Department of Medical Entomology, National Institute of Infectious Diseases, Tokyo, Japan; 5 Laboratory of Insect Genome Science, Faculty of Agriculture, Kyushu University, Fukuoka, Japan

**Keywords:** Influenza virus, Environmental microbiology, Entomology

## Abstract

High pathogenicity avian influenza (HPAI) poses a significant threat to both domestic and wild birds globally. The avian influenza virus, known for environmental contamination and subsequent oral infection in birds, necessitates careful consideration of alternative introduction routes during HPAI outbreaks. This study focuses on blowflies (genus *Calliphora*), in particular *Calliphora nigribarbis*, attracted to decaying animals and feces, which migrate to lowland areas of Japan from northern or mountainous regions in early winter, coinciding with HPAI season. Our investigation aims to delineate the role of blowflies as HPAI vectors by conducting a virus prevalence survey in a wild bird HPAI-enzootic area. In December 2022, 648 *Calliphora nigribarbis* were collected. Influenza virus RT-PCR testing identified 14 virus-positive samples (2.2% prevalence), with the highest occurrence observed near the crane colony (14.9%). Subtyping revealed the presence of H5N1 and HxN1 in some samples. Subsequent collections in December 2023 identified one HPAI virus-positive specimen from 608 collected flies in total, underscoring the potential involvement of blowflies in HPAI transmission. Our observations suggest *C. nigribarbis* may acquire the HPAI virus from deceased wild birds directly or from fecal materials from infected birds, highlighting the need to add blowflies as a target of HPAI vector control.

High pathogenicity avian influenza (HPAI) is an infectious disease caused by *alphainfluenzavirus influenzae* (Influenza A virus), represented by H5 or H7 subtype virus^1,2^. Numerous cases of HPAI infections in domestic and wild birds have been reported worldwide^2,3^. Particularly, the number of HPAI cases in Japan reached a record high in the 2022–2023 season. According to a report from the Japanese government agency, there were 242 cases in wild birds and 84 cases in poultry farms during that season, resulting in the culling of 17.7 million birds, despite significant efforts for prevention and control^4^. It is crucial to identify the pathways of infection for effective prevention.

The avian influenza virus is shed not only in respiratory droplets but also in feces. Excretion into feces can lead to viral contamination of the environment such as water pools, acting as a source of oral infection in other birds^5^. Therefore, careful attention is given to the introduction of contaminated materials, particularly bird excrement, into poultry houses. Measures such as disinfection of footwear and vehicles, as well as prevention of entry by small animals, are implemented. In cases where HPAI occurs despite these precautions, it is necessary to consider the possibility of alternative introduction routes.

Blowflies are well-known for their necrophagous habits, being attracted to deceased animals and birds to feed on decaying flesh. They are also attracted to feces, making them commonly observed insects around livestock facilities. Therefore, it is conceivable that blowflies acquire influenza viruses from the carrion of infected birds or excrement of infected birds. Indeed, HPAI viruses were detected in blowflies (*Callipholara nigribarbis*) collected in the vicinity of a poultry house where HPAI occurred with 20–30% virus-positive rates^6^. It was considered that *C. nigribarbis* uptakes viruses from dead poultry or their excrements.

While most species of blowflies, attracted by dead animals, are active in warm seasons, *C. nigribarbis* migrate to lowland areas from northern or mountainous regions in early winter and become active, corresponding to the HPAI flu season^7–11^. In laboratory conditions, the infectivity of influenza virus in *C. nigribarbis* was maintained within 24–48 hours^12^. *C. nigribarbis* can travel 1–2 km in two days^13^, suggesting that once a fly ingests the virus, it can propagate infectious viruses within a range of approximately 2 km. We investigated the potential role of blowflies as a vector of HPAI by conducting a survey on the virus prevalence in blowflies in a wild bird HPAI-endemic area.

We selected Izumi City in Kagoshima Prefecture, Japan, as our investigation site (Fig. 1A). Izumi City is renowned as a wintering site for cranes, with an annual observation of about 10,000 cranes, mainly *Gus monacha* and *Antigone vipio*. In the 2022–2023 season, 1600 cranes died due to HPAI, and there were successive cases of HPAI in poultry farms in the area (Fig. 1B). We established collection points in 10 sites to cover most areas of the city, including the crane colony and other areas rich in wild birds (*e.g.* riversides) (Fig. 1A). Blowflies were not collected around poultry farms (site K in Fig. 1) to comply with enzootic prevention measures. In December 2022, we collected a total of 755 flies, including 648 *C. nigribarbis* (Table 1). We dissected all the collected *C. nigribarbis* to test the presence of the influenza virus in their crops and intestines. As a result, we found 14 virus-positive samples among all collected *C. nigribarbis* (2.2% in total) in the real-time RT-PCR test. The virus prevalence was highest in the area near the crane colony (site B; 14.9%), and the virus was also detected in flies collected around the other two independent river mouths (site A; 1.4% and site D; 5.0%). This suggests that *C. nigribarbis* ingested viruses from infected cranes or other water birds such as ducks and coots. We also attempted to identify the subtype of the detected influenza virus with RT-PCR targeting H5 and N1 subtypes. We could identify two out of 14 samples as H5N1 subtypes (sample A1 and B3) and other two as HxN1 (sample B1 and B4) (Fig. 2). Unfortunately, we could not identify the subtypes of the other 10 samples with our primer sets, suggesting the degradation of viruses in the digestive tract of flies or the presence of other subtypes that were not targeted.

**Figure 1:**
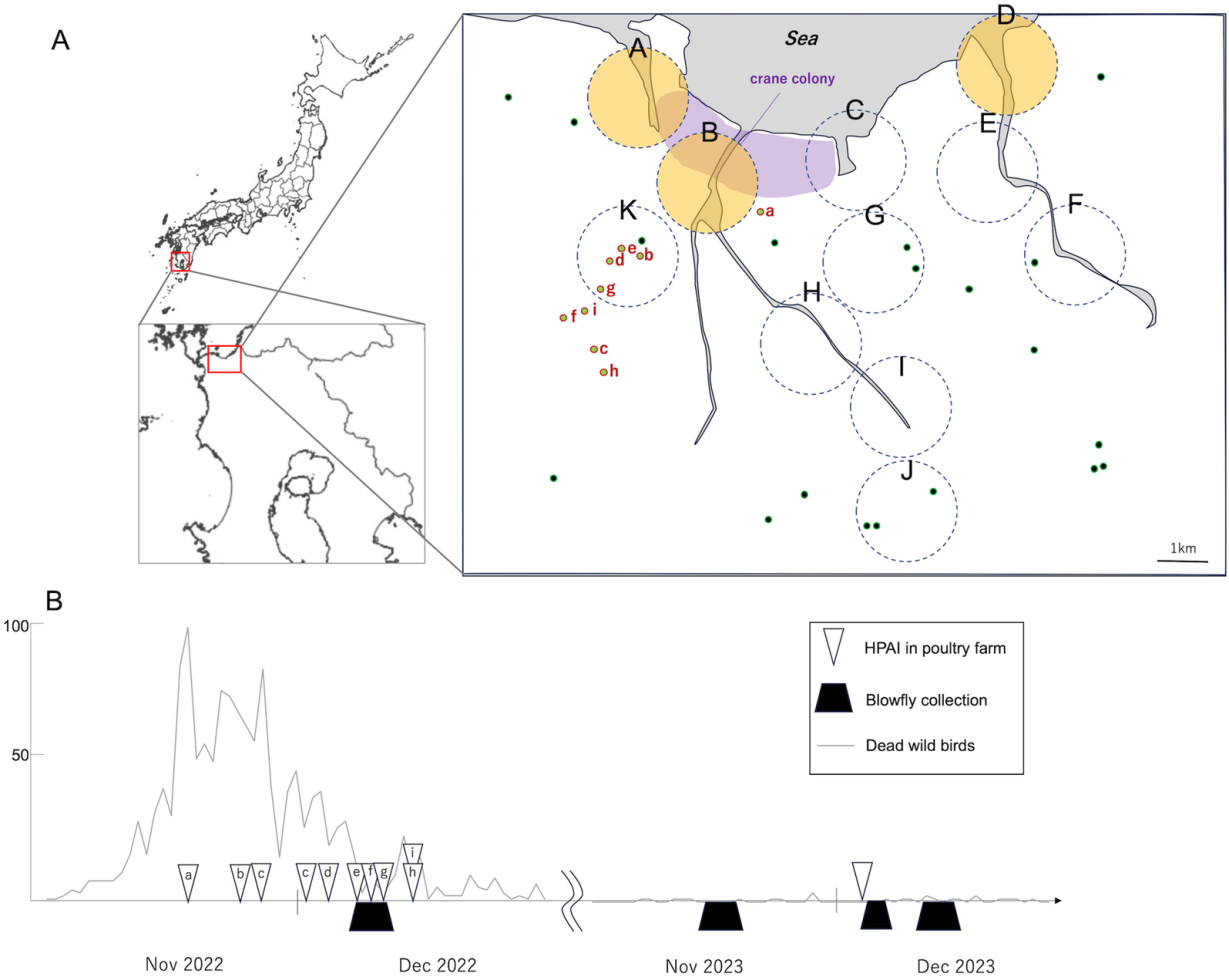
Blowfly collections and HPAI surveillance in Izumi City, Kagoshima, Japan. (**A**) Geographical view of Izumi City, Kagoshima, Japan, and the blowfly collections sites. Dashed circles (A–K; 1 km in radius) represent sites for blowfly collection (Orange: virus-detected). Green dots indicate the distribution of poultry farms, and light green dots labeled with alphabets (a–i) represent the farms with HPAI in 2022. The crane colony location is indicated in purple. (**B**) Schedule of blowfly surveillance. White arrowheads indicate the dates of HPAI cases in poultry farms. Black marks indicate the blowfly collection dates. The line graph depicts the number of dead wild birds in Izumi City, as reported by the Ministry of the Environment, Japan.

**Table 1:** Blowfly collection and virus detection in 2022 (7–9th Dec).

Site	No. of *C. nigribarbis*	No. of AIV-positive	Positive rate
A	146	2	1.4%
B	74	11	14.9%
C	90	0	0%
D	20	1	5.0%
E	81	0	0%
F	72	0	0%
G	34	0	0%
H	33	0	0%
I	52	0	0%
J	46	0	0%
K	–	–	–
Total	648	14	2.2%

**Figure 2:**
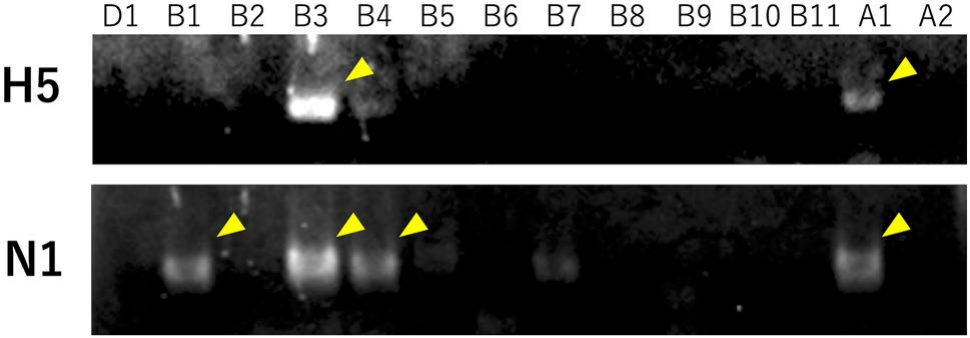
Influenza virus detection and RT-PCR analysis. RT-PCR analysis targeting the HA (H5) and NA (N1) genes of influenza virus in blowflies collected in 2022, which tested positive for the M gene. Samples indicated by yellow arrowheads were considered positive. Sample labels correspond to the site names in Fig. 1.

In the subsequent season, November 2023, we conducted blowfly surveys aiming for the early detection of HPAI (Table 2). We collected 208 *C. nigribarbis* at sites B, D, and K, but the influenza virus was not detected in this test. However, HPAI occurred in a poultry farm in early December, despite the prior negative detections from flies in this area. Two days after the occurrence, we revisited the site (sites B and K in Fig. 1) and collected flies, but no HPAI was detected among the 148 individuals of *C. nigribarbis* tested. Additionally, we conducted another round of blowfly collection in mid-December as part of continuous surveillance, collecting a total of 252 *C. nigribarbis* collected at sites A, B, D, and K. We identified one virus-positive specimen at site B with successful isolation of HPAI virus. Subsequent virus isolation experiments confirmed the infectivity of the detected virus, and sequencing analysis revealed that the subtype of the virus was H5N1 with the typical sequence of HPAI in the HA gene (PLREKRRKR/GLFG)^14^. The comparison of the isolated virus sequence with other HPAI viruses found in infected cranes revealed that the HA and NA gene sequences of the blowfly-derived HPAI virus were identical to those found in cranes collected two days before and 10 days after the blowfly collection. It is worth noting that the distance between the blowfly sampling location and the site where an HPAI-infected crane was found two days earlier is 800 m. When considered alongside surveillance conducted around poultry farms, our observations suggest that *C. nigribarbis* may acquire HPAI viruses from deceased wild birds but rarely from poultry, likely due to the swift culling of infected poultry.

**Table 2:** Blowfly collection and virus detection in 2023.

Collection date	site	No. of *C. nigribarbis*	No. of AIV-positive	Positive rate (%)
13–15th Nov	B, D, K	208	0	0
4–5th Dec	B, K	148	0	0
13–15th Dec	A, B, D, K	252	1	0.4

Blowflies represent a potential vector of HPAI, particularly in enzootic regions. The effectiveness of virus detection from flies relies heavily on the prevalence of infected and deceased wild birds. *C. nigribarbis* is widespread in human-populated areas across Japan, including semi-rural regions with poultry farms. Like other insects, *C. nigribarbis* intermittently disperses its feces, leading to environmental contamination. In this study, we focused on *C. nigribarbis* because it was the dominant blowfly species in our study field and the season, but we could not exclude the contribution of other necrophagous blowflies in HPAI propagation, especially in geographical areas.

Although the extent of blowfly intrusion into poultry houses and their role as infection sources has not been extensively studied, it is important to pay equal attention not only to the intrusion of small animals or birds but also to the entry of flies into poultry houses^15^. Unfortunately, due to the lack of comparable data on virus prevalence in each vector and their invasion rates into poultry farms, we could not determine which vector poses a higher risk for HPAI transmission on poultry farms.

Unlike house flies, which often originate within poultry houses and are visibly active, *C. nigribarbis* does not exhibit such behavior. While they may appear elusive, they can be readily captured in winter using baits or traps. Considering the possible involvement of blowflies in HPAI transmission, it would be advisable to implement fly control measures in poultry settings, such as utilizing fine mesh nets, fly traps, or insecticides.

## Materials and methods

### Fly collection, identification, and dissection

Blowflies were collected using the sweeping net method with decaying meat (horse or wild boar) or fish as bait. The collected flies were temporarily stored on ice for transportation (1–3 days) and then preserved at − 80 °C until further use. To ensure successful virus isolation, careful measures were taken to avoid freezing/thawing of the collected flies in 2023. Fly species were identified using morphological keys. Crops and intestines were excised from blowflies, separated into tubes, and then crushed in a shaker with zirconia beads (3000 rpm, 1 min). The samples were subsequently centrifuged (9000 rpm, 5 min), and the supernatants (homogenates) were harvested for further analysis.

### Virus detection

We adopted direct real-time RT-PCR for the detection of the *alphainfluenzavirus influenzae* M gene, following the procedures described elsewhere and NIID diagnostic manual for HPAI with some modifications^16^. The homogenated samples from flies were mixed with an equal amount of denaturing solution (20% Tween 20 and 0.04% SDS) and then incubated at 70 °C for 5 min. The denatured samples served as the template for real-time RT-PCR. We utilized the MP-29-57For and MP-183-153Rev primer set and MP-96-75ProbeAs as the probe. The reactions were prepared using PrimeScript One Step RT-PCR kit ver.2 (Takara) and analyzed in StepOnePlus real-time PCR system (Thermo Fisher Scientific).

HA and NA genes were identified by RT-PCR. The homogenates with an MP gene positive result were passed through the filter to remove debris, and then RNA was isolated from filtrates. The RT-PCR was carried out with primer sets targeting H5 subtype (5′-GAARCCTCTGATTTTRRAGGATTGTAG-3′ & 5′-TYTTGATAAGCCAYACCACATTTCTGA-3′) and N1 subtype (5′-TGGGCWRTAYACAGTAAGGACAA-3′ & 5′-ATWGTCAACCAACTGRTGCCATC-3′).

The influenza virus-positive specimen from December 2023 underwent further analysis at the National Institute of Animal Health, National Agriculture and Food Research Organization (Pathological Appraisal). In brief, the homogenate was inoculated into embryonated chicken eggs to confirm viral infectivity, and the sequence of HA and NA genes was determined. The resulting virus was designated as Influenza A virus (A/blow fly/Kagoshima/23a738D/2023, isolate ID: EPI_ISL_18969146).

Sequence data of HPAI virus in cranes (2023–2024 winter season, Kagoshima) were obtained from GISAID (EPI_ISL_18770565, EPI_ISL_18909437, EPI_ISL_18770564, EPI_ISL_18770563, EPI_ISL_18770562, and EPI_ISL_18651568) and used for sequence alignment and comparison.

### Supplementary Information

Supplementary Information 1.

Supplementary Information 2.

## Data Availability

Original data is provided within supplementary information files.
