# Supplementary figures and images for: Blowflies are potential vector for avian influenza virus at enzootic area in Japan
Source: Sci Rep. 2024 May 4;14:10285. doi: 10.1038/s41598-024-61026-1 (PMC11069500; doi:10.1038/s41598-024-61026-1)

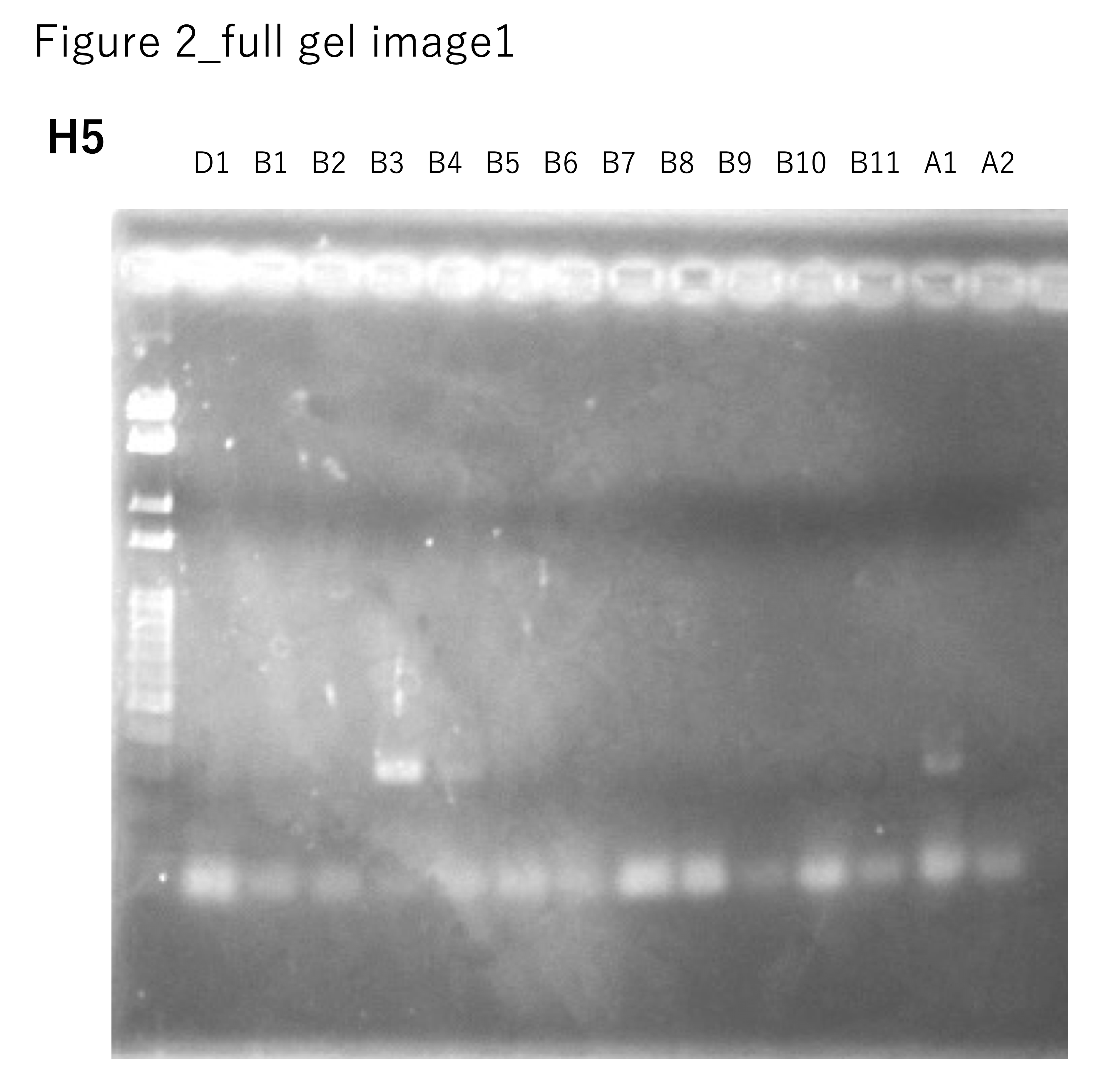

Supplement: Supplementary file 1 — Supplementary Information 1. [file 41598_2024_61026_MOESM1_ESM.tiff]

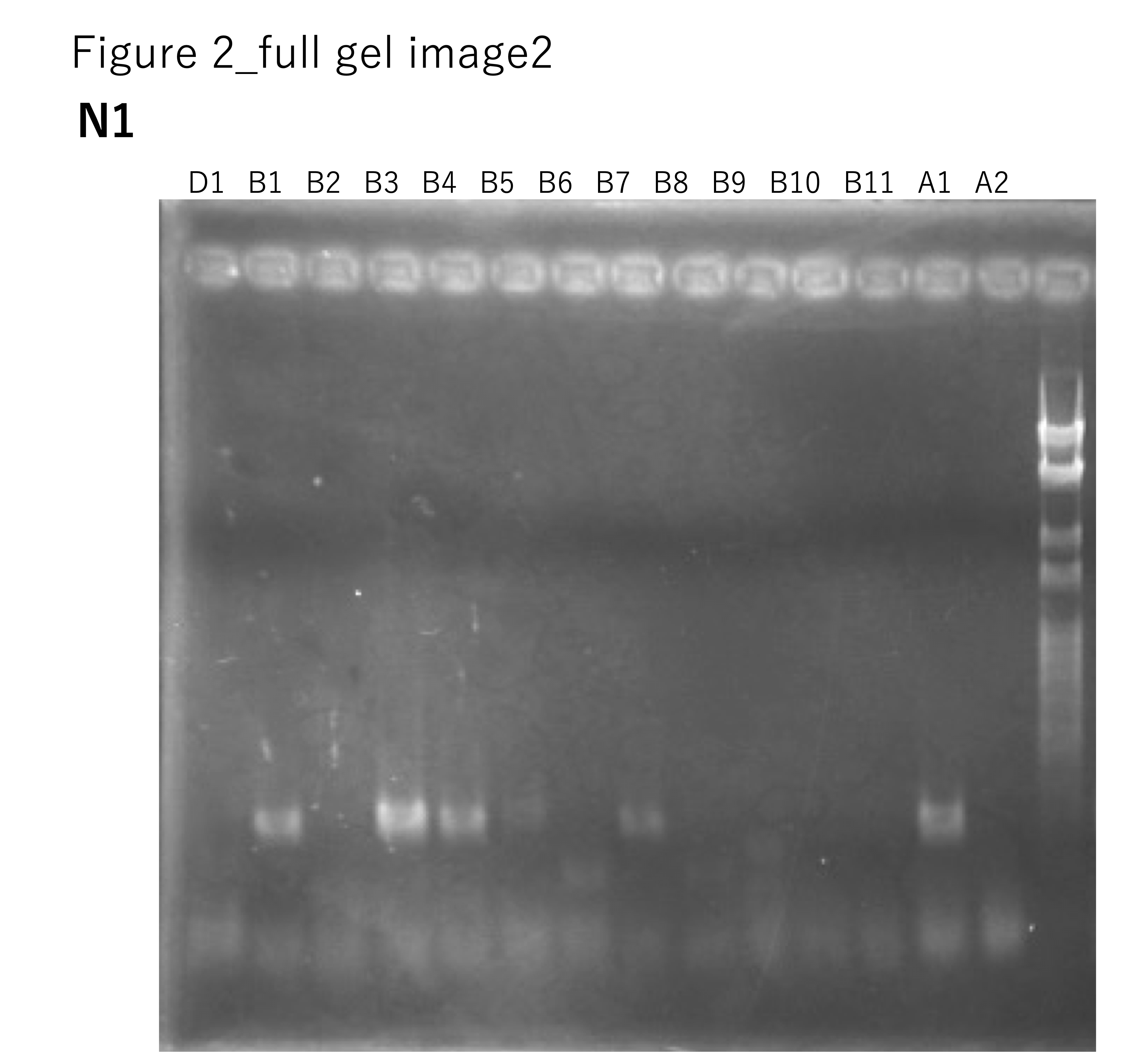

Supplement: Supplementary file 2 — Supplementary Information 2. [file 41598_2024_61026_MOESM2_ESM.tiff]
